# Supplementary material for: EnzML: multi-label prediction of enzyme classes using InterPro signatures
Source: BMC Bioinformatics. 2012 Apr 25;13:61. doi: 10.1186/1471-2105-13-61 (PMC3483700; doi:10.1186/1471-2105-13-61)
Supplement: Addtional file 5 — The Java code to format the data files, evaluate and predict. The file enzml_java_code.tar.gz contains the Java code used to format database data to ARFF and XML formats, to execute cross and train-test (jackknife) evaluations and to record evaluation results to database. More information is included in the readme.txt file and the Javadoc files. The code can be used with a MySQL database. To use a different database software, other JDBC drivers might be required. [file 1471-2105-13-61-S5.gz › java_code/enzml2011/doc/uk/ac/ed/inf/taweka/mulan/learn/database/MulanDbReader.html]

MulanDbReader


---


|  |  |  |
| --- | --- | --- |
| **Overview** \| **Package** \| **Class** \| **Use** \| **Tree** \| **Deprecated** \| **Index** \| **Help** | |  |
| **PREV CLASS**   **NEXT CLASS** | **FRAMES**    **NO FRAMES**     **All Classes** |  |
| SUMMARY: NESTED \| FIELD \| CONSTR \| METHOD | DETAIL: FIELD \| CONSTR \| METHOD |  |


---


## uk.ac.ed.inf.taweka.mulan.learn.database Class MulanDbReader

```
java.lang.Object
  uk.ac.ed.inf.utils.database.DbManaged
      uk.ac.ed.inf.utils.database.DbReader
          uk.ac.ed.inf.taweka.mulan.learn.database.MulanDbReader
```

---

``` public class MulanDbReader extends uk.ac.ed.inf.utils.database.DbReader ```

Reads ARFF file path from database

**Version:**
:   9 Jun 2010

**Author:**
:   Luna De Ferrari luna.deferrari-at-ed.ac.uk

---

**Constructor Summary**

`MulanDbReader(uk.ac.ed.inf.utils.database.DbManager dbManager)`


| **Method Summary** | |
| --- | --- |
| `java.lang.String` | `getArffPath(int id)`             Get an ARFF path from database row |

**Methods inherited from class uk.ac.ed.inf.utils.database.DbReader**

`executeQuery, getColumnValues, getDbMetaData, getListOfDbTables, getMap, getStatement, getTableReader, queryResultsColumnToCollection, queryResultsColumnToSet, queryResultsColumnToVector, tableExistsInDb`

**Methods inherited from class uk.ac.ed.inf.utils.database.DbManaged**

`getDbConnection, getDbManager`

**Methods inherited from class java.lang.Object**

`equals, getClass, hashCode, notify, notifyAll, toString, wait, wait, wait`

**Constructor Detail**

### MulanDbReader

```
public MulanDbReader(uk.ac.ed.inf.utils.database.DbManager dbManager)
```


**Method Detail**

### getArffPath

```
public java.lang.String getArffPath(int id)
```

:   Get an ARFF path from database row

    :   **Parameters:**: `id` - the record id of an ARFF file


---


|  |  |  |
| --- | --- | --- |
| **Overview** \| **Package** \| **Class** \| **Use** \| **Tree** \| **Deprecated** \| **Index** \| **Help** | |  |
| **PREV CLASS**   **NEXT CLASS** | **FRAMES**    **NO FRAMES**     **All Classes** |  |
| SUMMARY: NESTED \| FIELD \| CONSTR \| METHOD | DETAIL: FIELD \| CONSTR \| METHOD |  |


---
